# Mitochondrial Genome Analysis of *Isatis tinctoria* L. (Brassicaceae) Reveals Strengthened Purifying Selection Resulting From Recombination‐Driven Gene Duplication

**DOI:** 10.1002/ece3.72097

**Published:** 2025-09-10

**Authors:** Yongfu Li, Xiaoyun Hao, Yongjun Bi, Yayuan Zuo, Xiaoqin Sun

**Affiliations:** ^1^ Jiangsu Key Laboratory for Conservation and Utilization of Plant Resources, Institute of Botany Jiangsu Province and Chinese Academy of Sciences (Nanjing Botanical Garden Mem. Sun Yat‐Sen) Nanjing China; ^2^ Rural Energy and Environment Workstation of Ili Kazakh Autonomous Prefecture Yining China

**Keywords:** gene duplication events, *Isatis tinctoria* (Woad), mitochondrial genome, purifying selection

## Abstract

Plant mitochondrial genomes exhibit remarkable structural diversity and active recombination, yet their influence on the molecular evolutionary patterns of functional genes requires further empirical evidence across diverse lineages. Here, we assembled and annotated the complete mitochondrial genome of *Isatis tinctoria* L. (Brassicaceae), a phylogenetically informative species. The circular genome is 260,912 bp in length and contains 65 genes, including a pair of gene duplication events, *ccmB* and *trnK‐UUU*, situated between large direct repeats. Comparative analysis across 73 mitochondrial genomes—including 71 Brassicaceae accessions and 2 outgroups—revealed that genome size is significantly correlated with SSR abundance. This result supports the “repeat‐driven genome expansion” hypothesis. Using the *ccmB* gene as a representative, pairwise Ka/Ks analysis across 2850 species pairs revealed that Ka is significantly correlated with both Ks and GC content, suggesting that regional mutation rates and nucleotide composition jointly shape nonsynonymous substitution rates. Notably, species with *ccmB* gene copies displayed significantly lower Ka values than those without, whereas Ks values remained unchanged. This supports a “functional redundancy–purifying selection” model, in which gene duplication enhances system robustness and strengthens purifying selection on essential genes. Together, this study highlights the interplay among repeat architecture, recombination, and evolutionary constraint in the mitochondrial genome of *I. tinctoria* , and provides new insights into the structural evolution and selective dynamics of plant mitochondrial genomes.

## Graphical abstract

We believe our results provide important empirical evidence for the evolutionary consequences of genome structural variation in plants and offer new insights into the link between recombination, duplication, and molecular evolution in mitochondrial genomes.

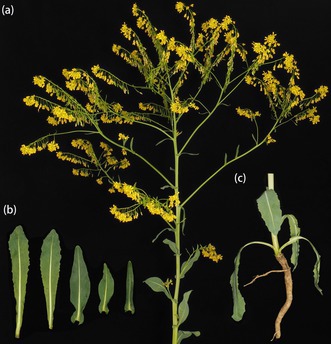

## 1 Introduction

Mitochondrial genomes of angiosperms are characterized by exceptional structural complexity and plasticity, with levels of variation far exceeding those observed in animals (Boore 1999; Ou et al. 2024). In contrast to animal mitochondrial genomes, which are typically compact and evolve rapidly, plant mitochondrial genomes span a broad size range—from approximately 200 kb to over 11 Mb—while maintaining remarkably low nucleotide substitution rates and highly conserved protein‐coding regions (Kan et al. 2022). This pronounced dichotomy—extensive structural dynamism alongside functional stability—makes plant mitochondrial genomes a compelling system for investigating the intrinsic relationships between genome architecture and molecular evolutionary dynamics (Christensen 2021).

Current models suggest that mitochondrial genome recombination in plants is driven by multiple interacting factors, including repeated sequences that serve as recombination substrates, DNA double‐strand break repair mechanisms, and coordinated regulation by nuclear‐encoded genetic pathways (Gualberto et al. 2014; Gualberto and Newton 2017). In recent years, several nuclear‐encoded DNA repair factors have been identified, particularly those involved in homologous recombination and mismatch repair. These pathways are essential for maintaining the stability and integrity of the mitochondrial genome (Odahara et al. 2015; Wallet et al. 2015). When such repair systems are impaired, error‐prone alternative mechanisms may become activated, leading to increased ectopic recombination, structural rearrangements, and elevated levels of heteroplasmy (Gualberto and Newton 2017).

Recombination activity also shows strong lineage specificity, with highly uneven rates across different evolutionary lineages (Alverson et al. 2011; Sullivan et al. 2020). For example, in the genus *Monsonia* L. (Geraniaceae), recombination rates vary by up to 600‐fold among lineages (Cole et al. 2018), while more than tenfold variation has been reported among genera in Rosaceae (Palmer et al. 2000). These recombination processes not only contribute to structural diversity but also influence gene function and evolutionary rates by affecting the accumulation and repair of mutations (Cheng et al. 2017; Kozik et al. 2019). Recent work has shown that mitochondrial genome copy number explains approximately 47% of the variation in synonymous substitution rates, suggesting that recombination may modulate evolutionary dynamics by altering the availability of repair templates (Zwonitzer et al. 2024). Additionally, lineage‐specific patterns of gene loss, intron gain, and substitution rates imply a close coupling between structural change and sequence evolution (Palmer et al. 2000; Møller et al. 2021).

Building on this background, whether structural rearrangements affect the rate of molecular evolution in functional regions has become a growing focus in plant mitochondrial genome research (Palmer and Herbon 1988; Sloan, Müller, et al. 2012; Skippington et al. 2015). While this question has been explored in model species such as *Arabidopsis thaliana* (L.) Heynh., systematic studies that integrate high‐quality genome assemblies with molecular evolutionary analysis remain limited in non‐model systems, particularly those with complex genomic structures and high levels of recombination.

In this study, we focus on *Isatis tinctoria* L., a representative species of Tribe Isatideae that holds particular phylogenetic importance within the Brassicaceae family (Moazzeni et al. 2010; Nikolov et al. 2019). Its placement in a relatively understudied lineage makes it a valuable taxon for comparative analyses of mitochondrial genome evolution. We generated a complete mitochondrial genome assembly using a combination of second‐ and third‐generation sequencing technologies and conducted comparative analyses with representative Brassicaceae species to identify structural features and potential repeat‐mediated recombination mechanisms. We further compared the substitution rates of the *ccmB* gene between species with and without gene duplication to assess whether recombination‐associated structural variation is linked to changes in molecular evolutionary rates. In addition to its scientific relevance, *I. tinctoria* is also the primary botanical source of the natural blue dye indigo and has been widely cultivated across Europe, Asia, and the Americas since the Middle Ages, contributing both economic and cultural significance (Speranza et al. 2020; Mocquard et al. 2022; Vauquelin et al. 2024). Characterizing the mitochondrial genome of *I. tinctoria* contributes to a better understanding of structural and evolutionary patterns in non‐model Brassicaceae species and offers a case study for examining potential links between recombination, mutation, and selection.

## 2 Materials and Methods

### 2.1 Sample Collection, Sequencing, Assembly and Annotation

**FIGURE 1:**
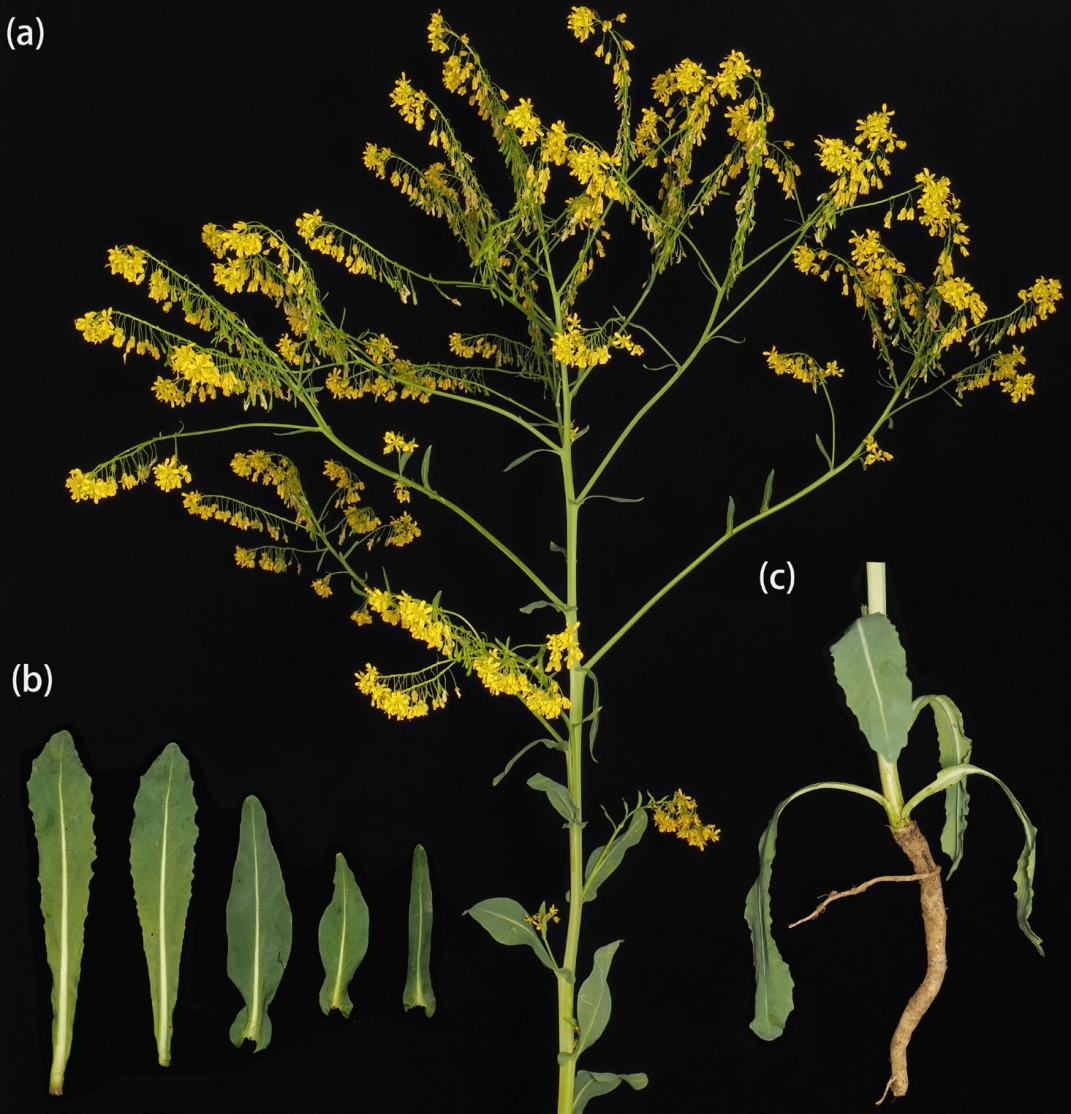
Morphological characteristics of *Isatis tinctoria* . (a) Aerial parts of the plant; (b) leaf morphology; (c) root system. Morphological images were photographed and generously provided by Dr. Yishuo Liang (Nanjing Botanical Garden, Mem. Sun Yat‐Sen).

Fresh leaves of *I. tinctoria* were collected from the experimental field at the Nanjing Botanical Garden MEM SUN YAT‐SEN (Figure 1). We employed a combination of second‐ and third‐generation sequencing strategies. The second‐generation sequencing followed Illumina's standard protocol, with data quality controlled using fastp (Chen et al. 2018), while third‐generation sequencing was performed on the Oxford Nanopore PromethION and filtered using filtlong (https://github.com/rrwick/Filtlong). The third‐generation data were aligned to a reference genome with minimap2 (Li 2018), corrected using Canu (Koren et al. 2017), and combined with the second‐generation data using bowtie2 (Langmead and Salzberg 2012). The final hybrid assembly was completed using the Unicycler pipeline (Wick et al. 2017), and visualized with Bandage (Wick et al. 2015) for manual adjustments. The assembled mitochondrial genome of *I. tinctoria* has been deposited in GenBank under accession number PV916015, providing a publicly accessible resource for future comparative and functional studies. The mitochondrial genome of *I. tinctoria* and other Brassicaceae species retrieved from NCBI were annotated using the PMGmap (Zhang et al. 2024).

### 2.2 Repetitive Sequence Identification

Three types of repeat sequences were investigated: Dispersed repeats (including forward, reverse, complement and palindromic), Simple sequence repeats (SSRs), and Tandem repeats. REPuter software (Kurtz and Schleiermacher 1999) was utilized to identify dispersed repeats, with parameters set to a Hamming distance threshold of 3. MISA‐web (Beier et al. 2017) was employed to detect SSRs, using thresholds of 10, 5, 4, 3, 3, and 3 for mono‐, di‐, tri‐, tetra‐, penta‐, and hexanucleotide repeats, respectively. Finally, Tandem Repeats Finder (Benson 1999) was used to identify tandem repeats, requiring a minimum repeat unit size of 7 base pairs.

### 2.3 Correlation Analysis of Mitochondrial Genome Features

Following the assembly and annotation of the *I. tinctoria* mitochondrial genome, we conducted a comparative analysis of mitochondrial genome features using 72 publicly available accessions retrieved from the NCBI database. Of these, 71 accessions represent 44 species (including subspecies and varieties) from 24 genera of Brassicaceae, while two outgroup species— *Batis maritima* L. (Bataceae) and *Carica papaya* L. (Caricaceae)—were included for comparative purposes, yielding a total of 73 mitochondrial genomes. To ensure consistent annotation across all taxa, the FASTA‐format genome sequences of published species were downloaded from NCBI and re‐annotated using the PMGmap pipeline (Zhang et al. 2024). To investigate patterns of mitochondrial genome variation and infer potential evolutionary associations, we constructed a data matrix summarizing key genomic features across all species, including genome size, repeat content, gene content, and gene duplication events. Pairwise correlation analyses were performed in R using the “cor()” function, and linear regression models were fitted with the “lm()” function to assess relationships among variables such as genome size, SSR abundance, and number of dispersed repeats. All results were visualized using the ggplot2 package to ensure clarity and reproducibility. Regression lines were plotted with 95% confidence intervals (shaded in light gray) using the geom_smooth (method = “lm”) function with default parameters. Group‐wise comparisons of genomic features (e.g., GC vs. AT content, Ka, Ks, and Ka/Ks ratios between duplicated and nonduplicated genes) were evaluated using the nonparametric Wilcoxon rank‐sum test (wilcox.test()). To assess structural differences and collinearity between the two available mitochondrial genome assemblies of *I. tinctoria* , we conducted pairwise genome alignment using Mauve v2.4.0 (Darling et al. 2004).

### 2.4 Phylogenetic and Molecular Substitution Rate Analysis

We used the same set of 73 mitochondrial genomes for phylogenetic reconstruction as in the correlation analysis. Due to substantial structural variation in mitochondrial genomes among species, direct multiple sequence alignment was not feasible. We extracted 23 conserved protein‐coding genes using PhyloSuite (Zhang et al. 2020) for phylogenetic reconstruction. Sequence alignment was performed using MAFFT (Katoh et al. 2002), and low‐quality regions were trimmed with trimAL (Capella‐Gutiérrez et al. 2009). ModelFinder identified “GTR + F + R2” as the optimal nucleotide substitution model. The phylogenetic tree was constructed using the maximum likelihood method in IQ‐TREE 2 (Minh et al. 2020), with 100,000 bootstrap replicates. Pairwise homologs of the *ccmB* gene were identified across 73 species and aligned using MAFFT. Nonsynonymous (Ka) and synonymous (Ks) substitution rates were estimated with KaKs_Calculator 2 (Wang et al. 2010), under the “YN” model. Correlation analyses among Ka, Ks, and guanine and cytosine (GC) content were performed in R using the “cor()” function. Boxplots were generated with the ggplot2 package to compare substitution rates between species with and without duplicated copies of the *ccmB* gene.

## 3 Results

**FIGURE 2:**
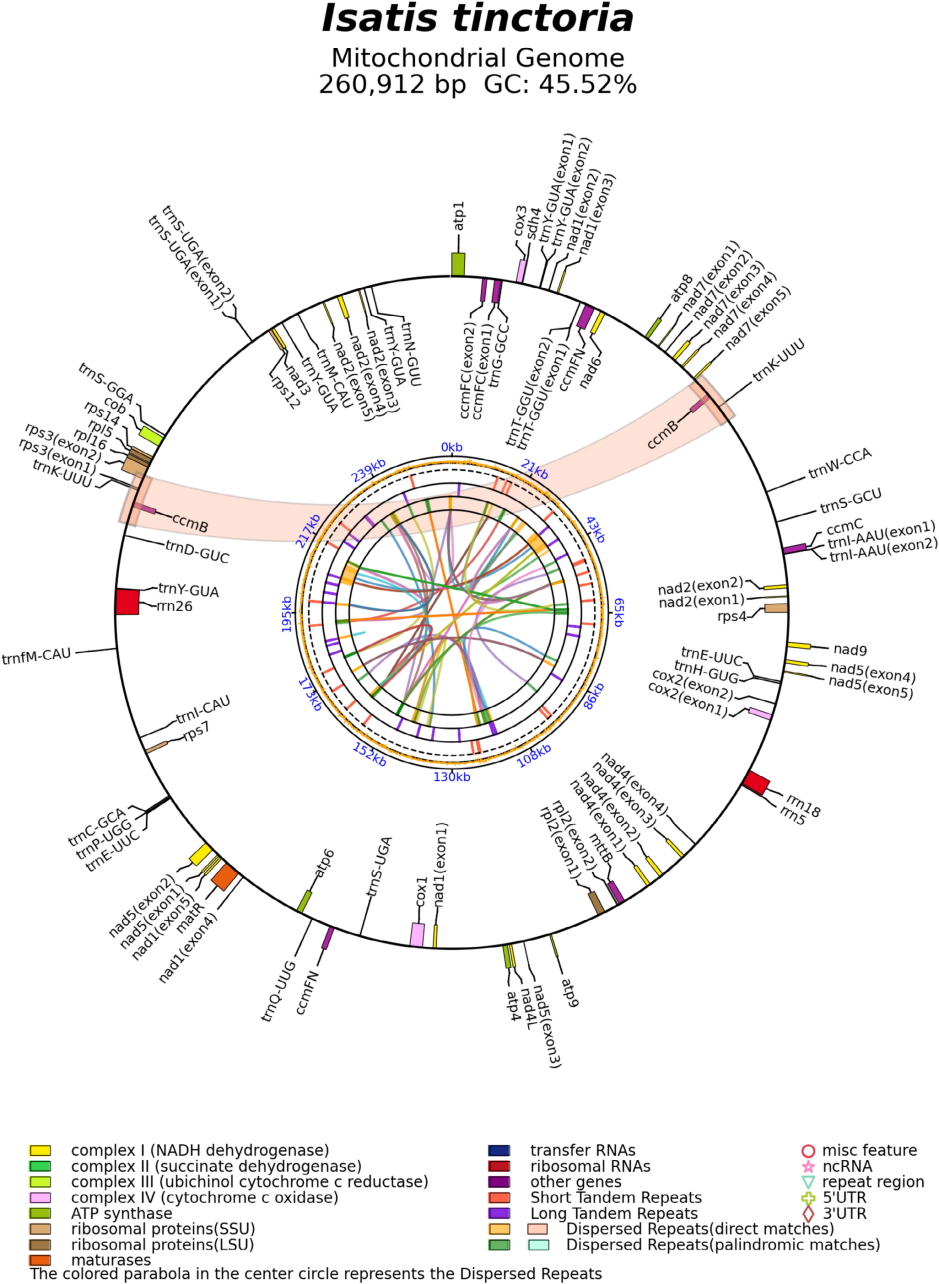
Assembly and annotated features of the mitochondrial genome of *Isatis tinctoria* . Circular map of the assembled mitochondrial genome showing gene annotations. A pair of large direct repeats is indicated by red connectors, with duplicated *ccmB* and *trnK‐UUU* genes positioned between direct repeats. *Note:* The circular structure is a hypothetical representation intended to facilitate genome annotation and visualization. It may not reflect the actual physical organization of the mitochondrial genome in vivo.

The mitochondrial genome of *I. tinctoria* assembled in this study is a single circular molecule with a total length of 260,912 bp and a GC content of 45.5% (Figure 2 and Table S1). A total of 65 genes were annotated, including 25 tRNA genes. Repetitive sequence analysis revealed 68 SSRs, 466 dispersed repeats (including 245 direct and 221 palindromic repeats), and 30 tandem repeats (Table S1). For comparison, a mitochondrial genome of *I. tinctoria* previously deposited in NCBI (accession: PP916044), which is 8990 bp shorter (251,922 bp), contains 64 annotated genes—one fewer than the 65 genes annotated in our assembly, which includes an additional copy of *trnY‐GUA*. The NCBI‐deposited genome contains 63 simple sequence repeats (SSRs), five fewer than in our assembly (68); 187 direct repeats (58 fewer), and 203 palindromic repeats (18 fewer). The total number of dispersed repeats is 390, which is 76 fewer than the 466 detected in our assembly. In addition, the number of tandem repeats is 26, four fewer than in our assembly (30). Global alignment of the two assemblies using Mauve identified five locally collinear blocks (LCBs) (Figure S1A). Three large insertions were identified in the newly assembled genome, including a 5155 bp insertion in LCB1, a 3380 bp insertion in LCB3, and a 265 bp insertion in LCB4 (Figure S1B–D). All insertions were primarily located in intergenic regions.

**FIGURE 3:**
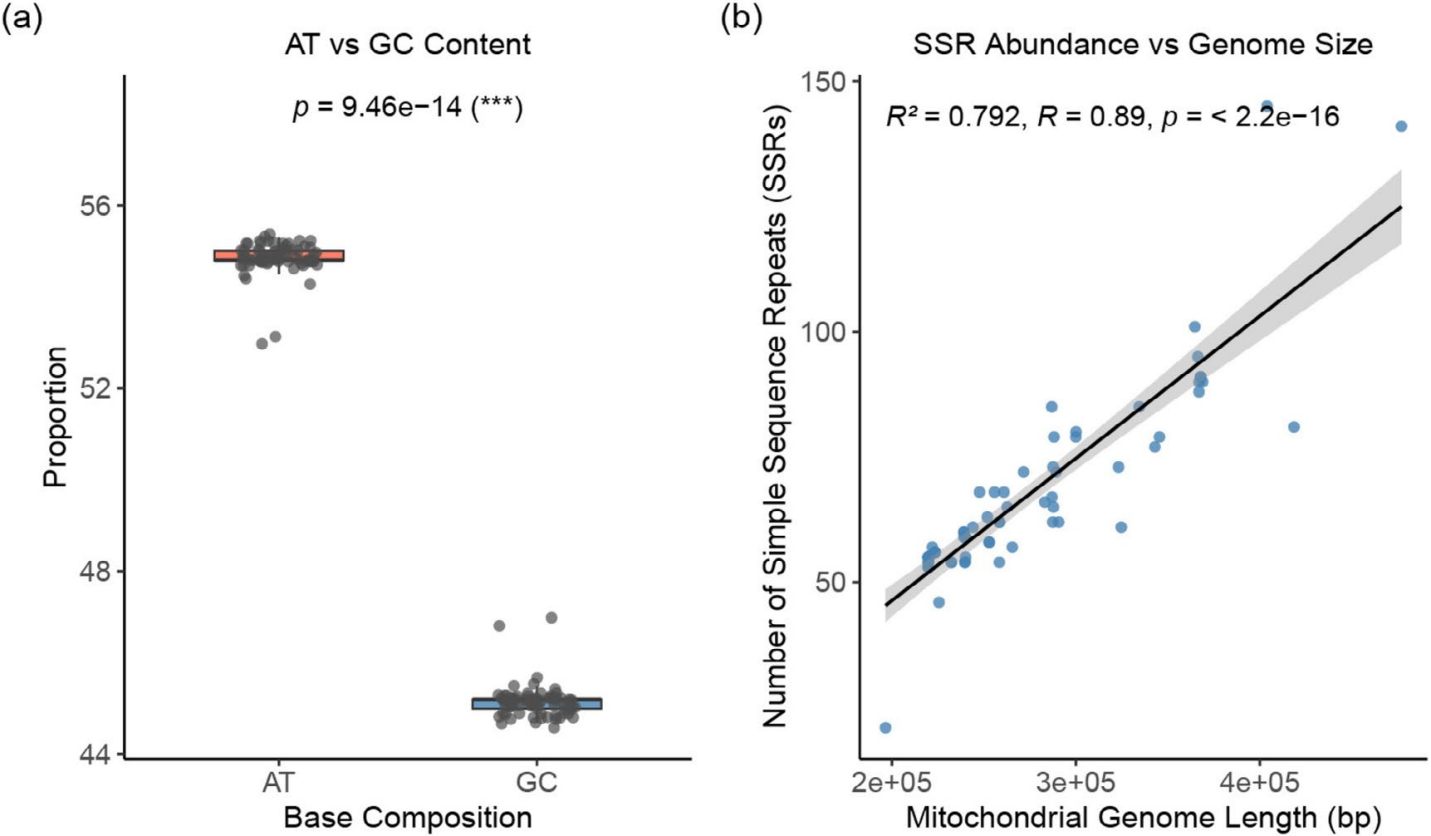
Statistical analyses of mitochondrial genome features across 73 species. (a) Boxplot comparing adenine–thymine (AT) and guanine–cytosine (GC) content. Statistical significance was assessed using Wilcoxon test. (b) Linear regression between total mitochondrial genome length and the number of simple sequence repeats (SSRs). Shaded gray areas around the regression lines represent 95% confidence intervals.

Statistical analyses based on 73 mitochondrial genomes were conducted to examine the correlations and evolutionary trends among various mitochondrial genome features. As shown in the boxplot (Figure 3A), AT content was significantly higher than GC content in the mitochondrial genomes (*p* = 9.46 × 10^−14^). A strong positive correlation exists between genome length and the number of SSRs (*R* ^2^ = 0.792, *R* = 0.89, *p* < 2.2 × 10^−16^; Figure 3B).

**FIGURE 4:**
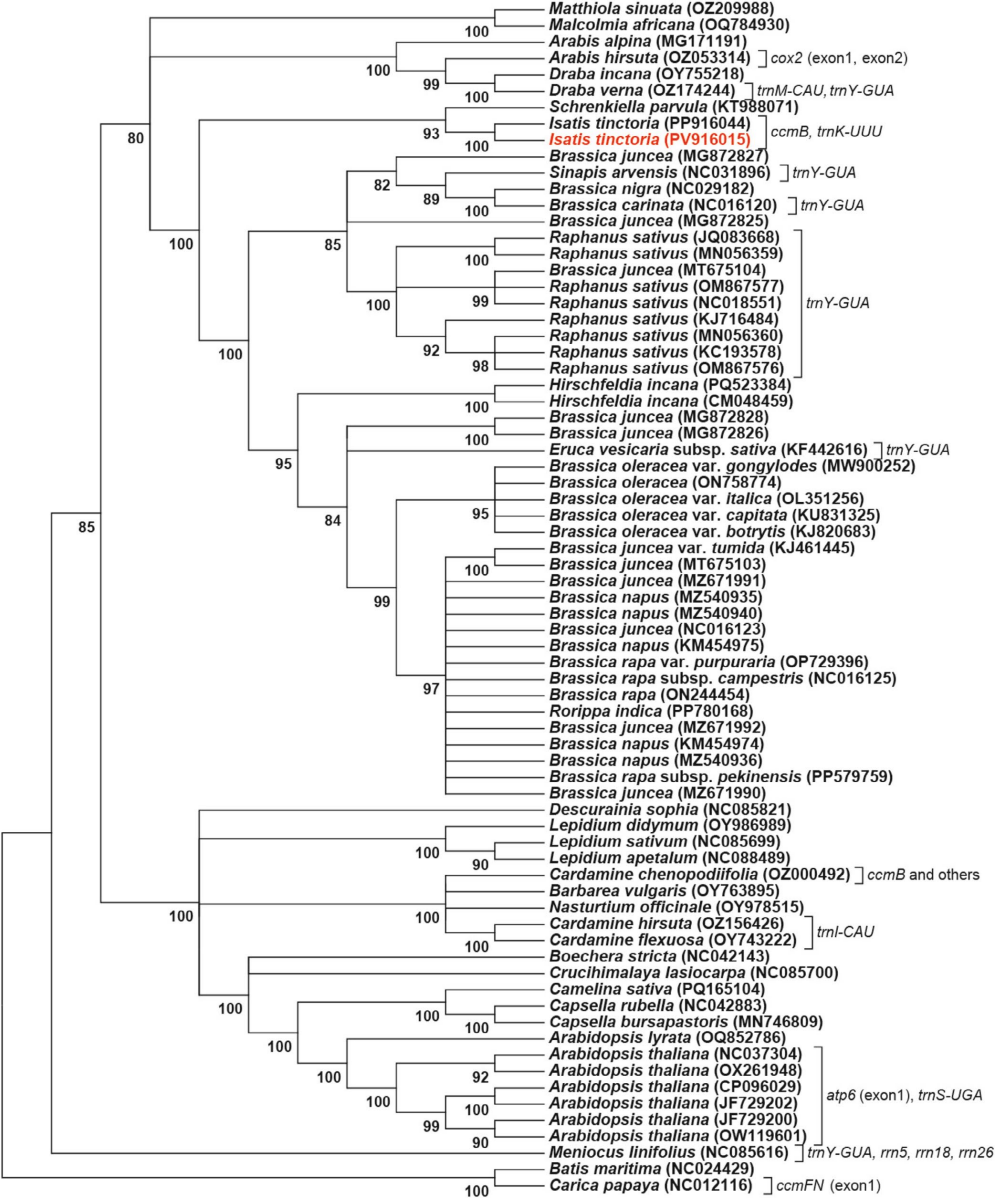
Maximum likelihood phylogenetic tree constructed from a concatenated alignment of 26 conserved protein‐coding genes shared among 73 accessions. Bootstrap support values are shown below each branch. Observed gene duplication events are annotated to the right of the corresponding species names; these events are likely to have arisen independently across different lineages.

A pair of gene duplication events, *ccmB* and *trnK‐UUU*, was identified in the mitochondrial genome of *I. tinctoria* . The mitochondrial genome map showed that both genes are located between a pair of direct dispersed repeats (Figure 2 and Figure S2). Comparative analysis based on phylogenetic relationships revealed that the gene duplication pattern observed in *I. tinctoria* is rare across Brassicaceae, with a *ccmB* gene copy detected in *Cardamine chenopodiifolia* (Figure 4). In contrast, *Schrenkiella parvula* (Schrenk) D.A. German and Al‐Shehbaz (KT988071), a closely related species, shows no evidence of mitochondrial gene duplication (Figure 4). Overall, gene duplication events in Brassicaceae mitochondrial genomes do not strictly follow phylogenetic constraints, and similar duplication patterns have occurred independently in multiple lineages (Figure 4). For example, *trnY‐GUA* is duplicated in several distantly related species, including *Raphanus raphanistrum* subsp. *sativus* (L.) Schmalh. (OM867577), *Mutarda arvensis* (L.) D.A. German (NC031896), *Meniocus linifolius* (Stephan ex Willd.) DC. (NC085616), *Eruca vesicaria* subsp. *sativa* (Mill.) Thell. (KF442616), *Draba verna* L. (OZ174244), *Brassica napus* L. (NC029182), *× Brassarda juncea* (L.) Su Liu and Z.‐H. Feng (MT675104), and *Brassica carinata* A. Braun (NC016120). In addition, several duplications were observed, such as *atp6* and *trnS‐UGA* in *A. thaliana* (CP096029), *cox2* in *Arabis hirsuta* (L.) Scop. (OZ053314), and *trnI‐CAU* in *× Brassica juncea* (MG872827), *Cardamine flexuosa* With. (OY743222), and *Cardamine hirsuta* L. (OZ156426). Furthermore, both *trnM‐CAU* and *trnY‐GUA* are duplicated in *D. verna* L. (OZ174244).

**FIGURE 5:**
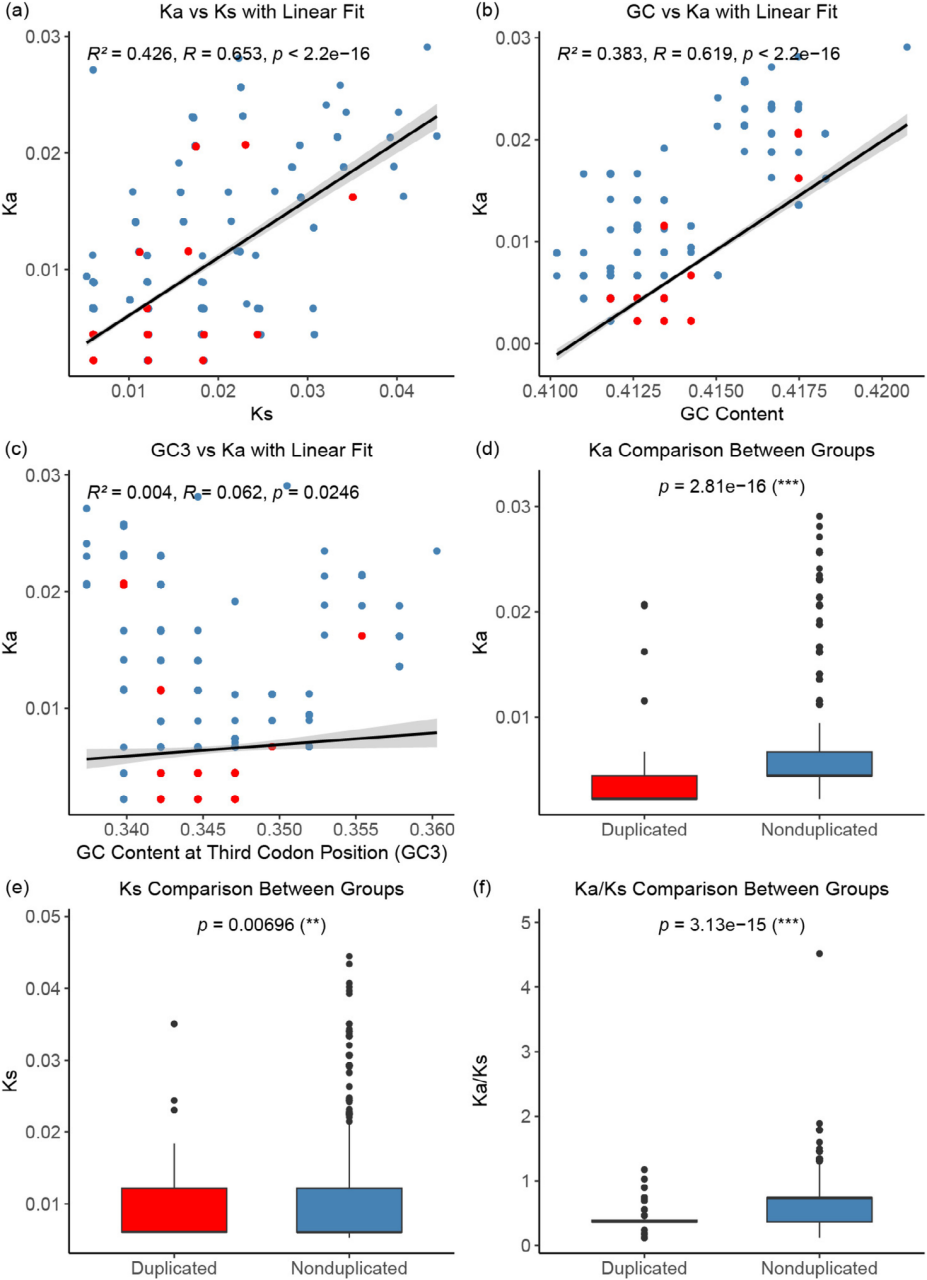
Molecular substitution rate analysis of the *ccmB* gene across 2850 pairwise combinations of 73 accessions, along with corresponding statistical evaluations. (a) Linear regression between the nonsynonymous substitution rate (Ka) and synonymous substitution rate (Ks). Shaded gray areas around the regression lines represent 95% confidence intervals. (b) Linear regression between GC content and Ka. (c) Linear regression between GC_3_ content and Ka. Red data points indicate values corresponding to species with duplicated *ccmB*. (d) Comparison of Ka values between species with and without *ccmB* gene copies. (e) Comparison of Ks values between species with and without *ccmB* gene copies. (f) Comparison of Ka/Ks values between species with and without *ccmB* gene copies. Statistical significance was assessed using Wilcoxon test.

To assess the evolutionary rate of the *ccmB* gene within recombination‐associated regions, we calculated pairwise Ka/Ks values across 73 accessions, resulting in 2850 species pairs. Statistical analyses revealed a significant positive correlation between Ka and Ks (*R* ^2^ = 0.426, *R* = 0.653, *p* < 2.2 × 10^−16^; Figure 5A). A similar pattern was observed between GC content and Ka (*R* ^2^ = 0.383, *R* = 0.619, *p* < 2.2 × 10^−16^; Figure 5B), whereas the correlation between the GC content in the third codon position (GC_3_) and Ka was negligible (*R* ^2^ = 0.004, *R* = 0.062, *p* = 0.0246; Figure 5C). Comparative analysis further showed that species with duplicated *ccmB* gene copies had significantly lower Ka values than those without duplication (*p* = 2.81 × 10^−16^; Figure 5D), while Ks values did not differ significantly between the two groups (*p* = 0.00696; Figure 5E). Moreover, Ka/Ks ratios were significantly lower in species with *ccmB* gene duplication compared to those without (*p* = 3.13 × 10^−15^; Figure 5F), suggesting stronger purifying selection associated with gene duplication.

## 4 Discussion

In this study, we assembled the complete mitochondrial genome of *I. tinctoria* , with a total length of 260,912 bp, which is 8990 bp longer than a previously deposited version in NCBI (accession: PP916044). The newly assembled genome contains one more annotated gene, corresponding to an additional copy of the tRNA gene *trnY‐GUA*, highlighting its structural variation. Intraspecific variation in mitochondrial genome size is a common feature among angiosperms. For instance, in *Silene vulgaris* (Moench) Garcke, mitochondrial genomes range from 361 to 429 kb among individuals, with only about half of the sequence content shared between any two genomes (Sloan, Müller, et al. 2012). Similar variation has also been observed within Brassicaceae, where accessions of *A. thaliana* (Arrieta‐Montiel et al. 2009) and *B. napus* (Chang et al. 2011) exhibit notable differences in mitochondrial genome size. Such structural variation is often driven by homologous recombination associated with double‐strand break repair, along with the proliferation of repeated sequences (Davila et al. 2011; Sanchez‐Puerta et al. 2015). In our assembly, global alignment with the NCBI‐deposited genome revealed large insertions in several regions (Figure S1), primarily within intergenic sequences. In addition to biological factors, the structural differences observed between the two assemblies may also reflect discrepancies arising from the use of different assembly strategies. To better characterize structural variation within *I. tinctoria* , future studies incorporating population‐level sampling are recommended.

Comparative analysis across 73 accessions revealed a significant positive correlation between mitochondrial genome length and the number of SSRs. This result supports the “repeat‐driven genome expansion” hypothesis. This model proposes that repetitive elements, by serving as templates for recombination, not only facilitate structural rearrangements but also promote genome expansion through mechanisms such as non‐allelic homologous recombination or replication slippage (Davila et al. 2011; Kong et al. 2020; Xia et al. 2020).

In the mitochondrial genome of *I. tinctoria* , two large direct repeats were identified, each containing a copy of *ccmB* and *trnK‐UUU*, indicating that these genes are duplicated as part of a repeat‐mediated structural rearrangement (Figure 2). Assembly graph analysis further supports the possibility that these gene duplications arose via homologous recombination mediated by the flanking repeats (Figure 2). Phylogenetic comparison shows that this duplication pattern is absent in the closely related species *S. parvula* and appears to be rare across the Brassicaceae family, indicating that the duplication likely originated independently in *I. tinctoria* .

Comparative analysis further revealed that gene duplication events in Brassicaceae mitochondrial genomes do not strictly follow phylogenetic relationships (Figure 4). Several duplications appear to have arisen independently in distantly related taxa—for example, *trnY‐GUA* has been duplicated in *B. napus* , *Raphanus sativus* , *M. arvensis* (L.) D.A. German, and *M. linifolius*. This suggests that gene duplication is more likely driven by local structural features, such as the spatial distribution of repeat sequences, the location of recombination hotspots, and the intensity of recombination activity, rather than being solely determined by shared evolutionary ancestry.

Gene duplication is often closely associated with recombination hotspots—regions of the genome where crossover events occur at elevated frequencies. In plant genomes, such hotspots are frequently located near regulatory elements, and their activity can facilitate gene duplication and diversification (Brazier and Glémin 2024). These recombination‐mediated events are typically associated with dispersed repeat sequences, which can trigger non‐allelic homologous recombination (NAHR) and lead to the duplication of genes or genomic segments (Xia et al. 2020; Li et al. 2021).

In contrast, some gene duplications display clear lineage specificity. For example, *atp6* and *trnS‐UGA* duplications are exclusively observed in the species *A. thaliana* (Marienfeld et al. 1996), while the duplication of *cox2* has only been observed in *A. hirsuta* . Additionally, *trnI‐CAU* and *trnY‐GUA* duplications are concentrated in members of the genus *Cardamine* L., suggesting that these events may be linked to lineage‐specific recombination mechanisms, regulatory landscapes, or ecological adaptations. In some cases, such structural variations have been shown to correlate with phenotypic traits such as cytoplasmic male sterility (CMS), highlighting their potential functional relevance (Verhage 2020; Wang et al. 2020). Integrating comparative genomic data with functional genomics and population‐level analyses may help clarify whether lineage‐specific duplications confer adaptive advantages or reflect responses to specific selective pressures.

Although the duplication of *ccmB* and *trnK‐UUU* appears to be unique to *I. tinctoria* within the sampled Brassicaceae taxa, this conclusion is based on phylogenetic inference derived from concatenated mitochondrial coding sequences. However, such coding regions may not fully capture the evolutionary history of structural features, particularly those mediated by repetitive elements. As plant mitochondrial genomes are known to undergo parallel duplications and exhibit occasional horizontal gene transfer, the observed duplications may reflect homoplastic or convergent events rather than strict species specificity (Bergthorsson et al. 2004; Mower et al. 2010; Christensen 2013; Garcia et al. 2021). Further comparative analyses using broader taxon sampling and synteny‐based alignments will be needed to clarify the evolutionary origins of these duplications.

To further investigate whether structural rearrangements in the mitochondrial genome influence the molecular evolutionary rates of functional genes, we used *ccmB* as a representative gene and conducted pairwise Ka/Ks analyses across 2850 combinations of 73 mitochondrial genomes. The results revealed a moderately strong to strong positive correlation between Ka and Ks (*r* = 0.653, *R* ^2^ = 0.426), and a moderately strong correlation between Ka and overall GC content (*r* = 0.619, *R* ^2^ = 0.383) (Figure 5A–B), suggesting that variation in nonsynonymous substitution rates is shaped by both local mutation dynamics and nucleotide composition bias. These findings are broadly consistent with previous studies proposing that base composition and regional mutation processes jointly modulate substitution rates in plant mitochondrial genomes (Sloan and Wu 2014). However, GC_3_ content showed a negligible correlation with Ka (*r* = 0.062, *R* ^2^ = 0.0038), despite statistical significance (*p* = 0.0246), suggesting that the observed association with GC content likely reflects broader sequence composition rather than codon position bias or GC‐biased gene conversion. This further highlights the role of genome‐wide context, such as local mutational environments or structural constraints, in shaping evolutionary rate variation (Sloan, Alverson, et al. 2012).

Several theoretical models have been proposed to explain how gene duplication affects the molecular evolution of coding sequences. The neofunctionalization hypothesis suggests that relaxed selective constraints on one copy may facilitate the acquisition of novel functions, often accompanied by accelerated nonsynonymous substitution rates (Costello et al. 2020). In contrast, the subfunctionalization model posits that duplicated genes may partition ancestral functions, leading to complementary retention and mild relaxation of constraint on each copy (Force et al. 1999). A third framework, the dosage balance hypothesis, emphasizes the retention of duplicate genes without major functional divergence, under stabilizing selection to maintain transcript abundance and stoichiometric balance (Birchler and Veitia 2007).

However, our findings are most consistent with the functional redundancy–purifying selection model (Crow and Wagner 2005; Hsiao and Vitkup 2008). Notably, species harboring duplicated copies of *ccmB* exhibited significantly lower Ka values (Figure 5C) and reduced Ka/Ks ratios (Figure 5F) compared to species with single‐copy *ccmB*, whereas Ks values did not differ significantly between the two groups (Figure 5D). This pattern supports the idea that gene duplication enhances genetic robustness and allows nonsynonymous mutations to be more effectively purged by purifying selection. In the context of the structurally dynamic and recombinationally active plant mitochondrial genome, such a mechanism may be particularly important for maintaining the integrity of essential genes. The *ccmB* gene, which is involved in cytochrome c biogenesis, appears to be under especially strong functional constraint in its duplicated form, likely to ensure stable expression and mitochondrial respiratory efficiency. While the current study does not include transcriptomic data, future research incorporating gene expression and functional analyses could help elucidate the regulatory consequences and selective advantages of gene duplication events in *I. tinctoria* and related species.

## 5 Conclusion

This study provides a comprehensive analysis of the structural organization and evolutionary characteristics of the mitochondrial genome of *I. tinctoria* . The newly assembled genome spans 260,912 bp and contains 65 annotated gene copies, including a pair of duplicated genes *ccmB* and *trnK*‐*UUU*, positioned between a set of large direct repeats. The widespread presence of SSRs, direct repeats offers physical substrates for genome rearrangement and gene duplication, reflecting a high degree of structural plasticity in this mitogenome.

Comparative analyses revealed significant correlations between mitochondrial genome length and SSR abundance, highlighting the critical role of repeated sequences in shaping mitochondrial genome structure. Within a phylogenetic framework, the duplications of *ccmB* were observed in *I. tinctoria* and also in *C*. *chenopodiifolia* (OZ000492), a phylogenetically distant species within Brassicaceae. Although the structural details of these duplications are not entirely identical, their independent occurrence suggests that such events are more likely driven by lineage‐specific recombination dynamics and local structural contexts than by shared ancestry. Moreover, multiple other gene duplication events were also found to arise independently across distinct lineages, supporting the view that repeat‐mediated duplications in plant mitochondrial genomes frequently reflect parallel evolutionary trajectories rather than inherited traits.

Further analysis of substitution rates showed that Ka values for *ccmB* were significantly positively correlated with both Ks and GC content, indicating that local mutation rates and nucleotide composition jointly influence molecular evolutionary dynamics. Notably, species carrying *ccmB* gene copies exhibited significantly lower Ka values, while Ks values remained unchanged, consistent with the “functional redundancy–purifying selection” model. This suggests that gene duplication enhances selective constraint, contributing to the maintenance of essential gene function and expression.

Collectively, our findings reveal a tightly coupled relationship among repeat architecture, recombination activity, and molecular evolution in the *I. tinctoria* mitochondrial genome. These results provide valuable insights into the structural evolution of plant mitochondrial genomes and offer a new perspective on how duplication, recombination, and selection collectively shape plant mitochondrial genome evolution.

## Author Contributions

**Yongfu Li:** conceptualization (equal), data curation (equal), formal analysis (equal), funding acquisition (equal), investigation (equal), methodology (equal), project administration (equal), resources (equal), software (equal), validation (equal), visualization (equal), writing – original draft (equal), writing – review and editing (equal). **Xiaoyun Hao:** formal analysis (equal), investigation (equal), software (equal). **Yongjun Bi:** investigation (equal). **Yayuan Zuo:** formal analysis (equal), investigation (equal). **Xiaoqin Sun:** conceptualization (equal), funding acquisition (equal), methodology (equal), project administration (equal), supervision (equal), validation (equal).

## Conflicts of Interest

The authors declare no conflicts of interest.

## Supporting information


**Figure S1:** MAUVE alignment between the newly assembled mitochondrial genome of *Isatis tinctoria* (PV916015) and a previously submitted version (PP916044) available in NCBI. (a) Alignment result showing five locally collinear blocks (LCBs) identified by MAUVE between the two accessions; (b) A 5155 bp insertion in PV916015 within LCB1; (c) A 3380 bp insertion in PV916015 within LCB3; (d) A 265 bp insertion in PV916015 within LCB4.

**FIGURE S2:** The raw assembly graph showing four pairs of long dispersed repeats: 9 and 9_copy, 10 and 10_copy, 11 and 11_copy, and 12 and 12_copy. *Note:* The circular structure is a hypothetical representation intended to facilitate genome annotation and visualization. It may not reflect the actual physical organization of the mitochondrial genome in vivo.

**Table S1:** Summary of genome features and repeat content for 73 mitochondrial accessions. This table provides detailed information for each of the 73 mitochondrial genome accessions analyzed in this study. Included are the species name, total genome length in base pairs (length), and base composition in terms of GC and AT content (%). The Gene and tRNA columns indicate the total number of annotated protein‐coding genes and transfer RNA genes, respectively. The SSR_count column refers to the number of simple sequence repeats identified using MISA‐web. Repeat structures were further annotated using REPuter and Tandem Repeats Finder: D_count represents the number of forward (direct) dispersed repeats, while P_count indicates palindromic dispersed repeats; the sum of these two is given in Dispersal_repeats. Tandem_count represents the total number of tandem repeats identified with Tandem Repeats Finder. All annotations were generated using a standardized pipeline across all accessions.

## Data Availability

The complete mitochondrial genome sequence of *I. tinctoria* generated in this study has been deposited in GenBank under accession number PV916015.
