# Supplementary material for: Mitochondrial Genome Analysis of Isatis tinctoria L. (Brassicaceae) Reveals Strengthened Purifying Selection Resulting From Recombination‐Driven Gene Duplication
Source: Ecol Evol. 2025 Sep 10;15(9):e72097. doi: 10.1002/ece3.72097 (PMC12423112; doi:10.1002/ece3.72097)
Supplement: Supplementary file 1 — Figure S1: MAUVE alignment between the newly assembled mitochondrial genome of Isatis tinctoria (PV916015) and a previously submitted version (PP916044) available in NCBI. (a) Alignment result showing five locally collinear blocks (LCBs) identified by MAUVE between the two accessions; (b) A 5155 bp insertion in PV916015 within LCB1; (c) A 3380 bp insertion in PV916015 within LCB3; (d) A 265 bp insertion in PV916015 within LCB4. [file ECE3-15-e72097-s001.docx]

**(a)**


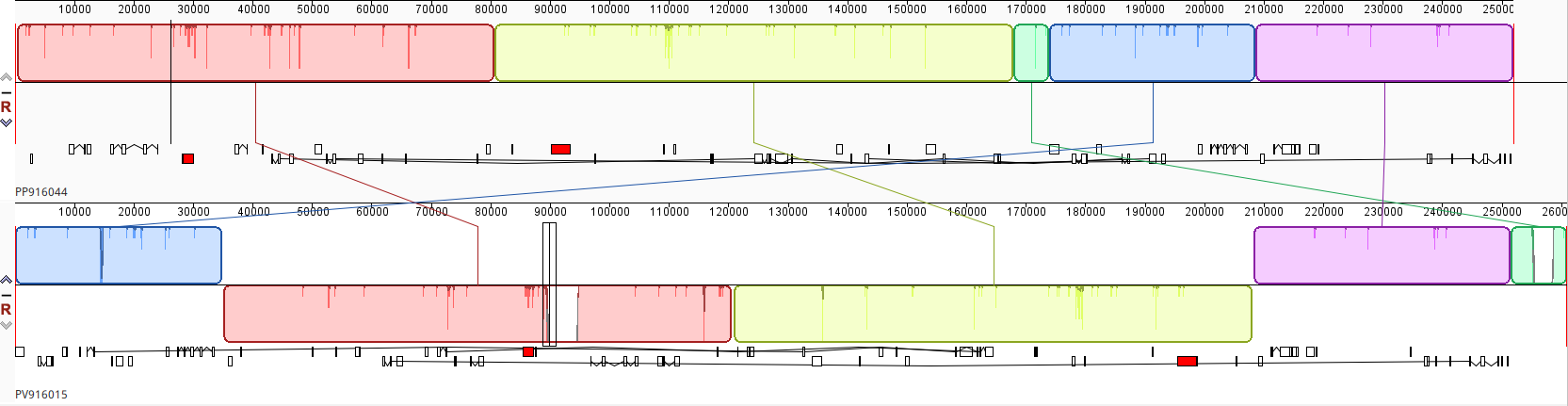


**(b)**


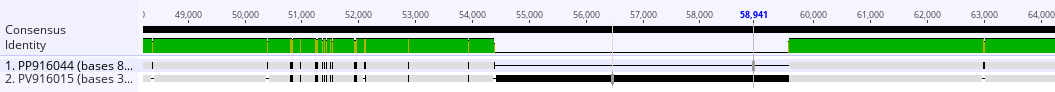


**(c)**


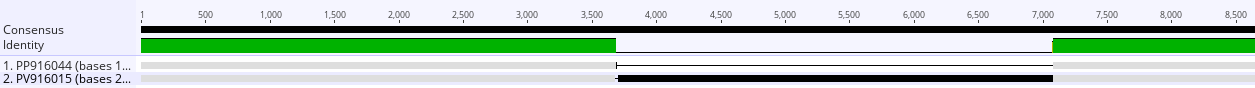


**(d)**


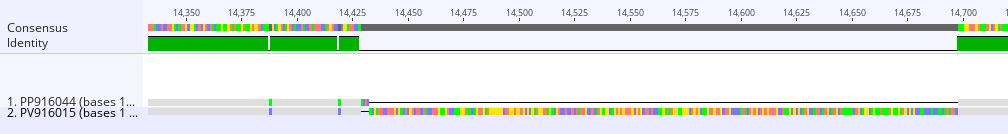


**FIGURE S1**. MAUVE alignment between the newly assembled mitochondrial genome of *I. tinctoria* (PV916015) and a previously submitted version (PP916044) available in NCBI. (a) Alignment result showing five locally collinear blocks (LCBs) identified by MAUVE between the two accessions; (b) A 5,155 bp insertion in PV916015 within LCB1; (c) A 3,380 bp insertion in PV916015 within LCB3; (d) A 265 bp insertion in PV916015 within LCB4.
