# Supplementary material for: Mitochondrial Genome Analysis of Isatis tinctoria L. (Brassicaceae) Reveals Strengthened Purifying Selection Resulting From Recombination‐Driven Gene Duplication
Source: Ecol Evol. 2025 Sep 10;15(9):e72097. doi: 10.1002/ece3.72097 (PMC12423112; doi:10.1002/ece3.72097)
Supplement: Supplementary file 2 — FIGURE S2: The raw assembly graph showing four pairs of long dispersed repeats: 9 and 9_copy, 10 and 10_copy, 11 and 11_copy, and 12 and 12_copy. Note: The circular structure is a hypothetical representation intended to facilitate genome annotation and visualization. It may not reflect the actual physical organization of the mitochondrial genome in vivo. [file ECE3-15-e72097-s003.docx]

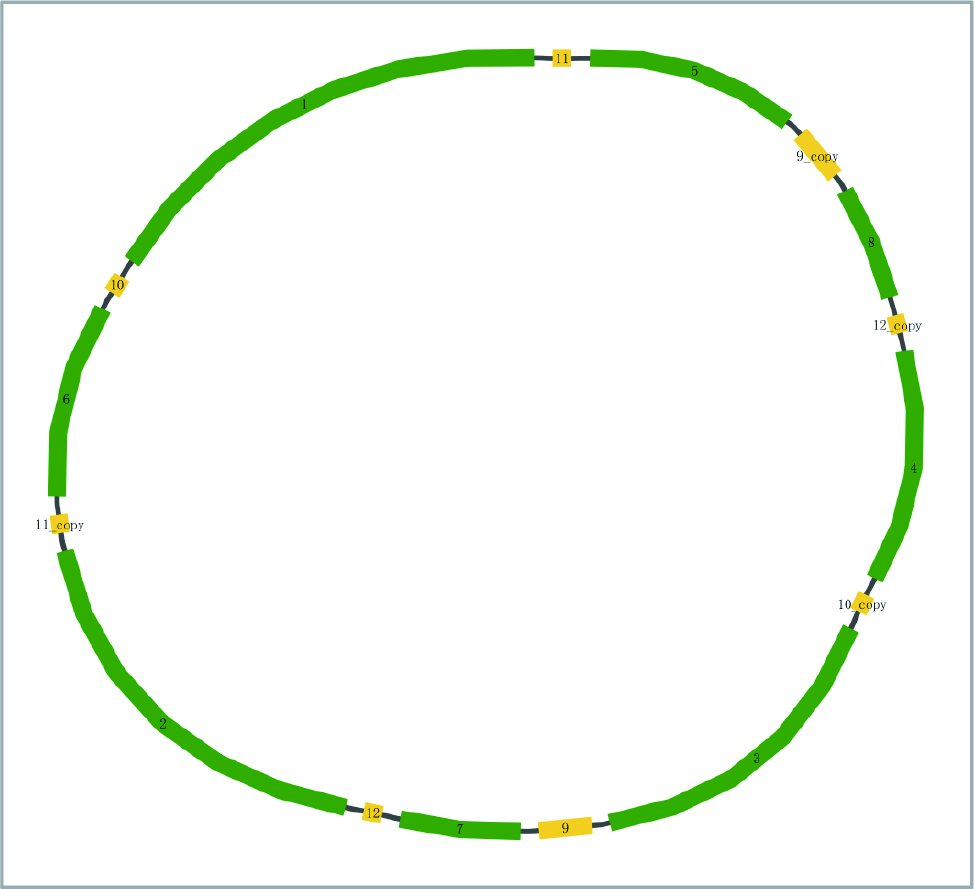


FIGURE S2 The raw assembly graph showing four pairs of long dispersed repeats: 9 and 9_copy, 10 and 10_copy, 11 and 11_copy, and 12 and 12_copy. Note: The circular structure is a hypothetical representation intended to facilitate genome annotation and visualization. It may not reflect the actual physical organization of the mitochondrial genome in vivo.
